# Turmeric–Black Cumin Essential Oils and Their Capacity to Attenuate Free Radicals, Protein Denaturation, and Cancer Proliferation

**DOI:** 10.3390/molecules29153523

**Published:** 2024-07-26

**Authors:** Ayodeji Oluwabunmi Oriola

**Affiliations:** Department of Chemical and Physical Sciences, Walter Sisulu University, Mthatha 5117, South Africa; aoriola@wsu.ac.za; Tel.: +27-65-593-4742

**Keywords:** turmeric, black cumin, essential oils, antioxidant, protein denaturation, anticancer

## Abstract

Turmeric rhizomes (*Curcuma longa*) and black cumin seeds (*Nigella sativa*) are polyherbal ingredients used for the management of cancer and other chronic inflammatory diseases in Nigerian ethnomedicine. Previous studies have shown the antioxidant, anti-inflammatory, and anticancer activities of the individual plant extracts. However, the two spices have not been biologically potentiated in their combined form. Therefore, this study obtained essential oils (EOs) from the combined spices and evaluated their inhibitory effects on free radicals, protein denaturation, and cancer proliferation. The EOs were extracted by hydro-distillation (HD) and characterized by gas chromatography-mass spectrometry (GC-MS). In vitro antioxidant assessment was conducted based on DPPH, hydrogen peroxide (H_2_O_2_), nitric oxide (NO), and ferric ion (Fe^3+^) radical scavenging assays. The cytotoxicity of the oil against non-tumorigenic (HEK293) and cancerous (HepG2 and HeLa) cell lines was determined following the MTT cell viability assay. An in silico molecular docking analysis of the oil constituents was also performed. Six batches of EOs I–VI were afforded, comprising twenty-two major constituents, with aromatic *Ar*-turmerone being the most prominent compound. There was a marked improvement in the bioactivity of the oils upon repeated HD and as a combination. The batch VI oil exhibited the best activity, with a cytotoxicity (CC_50_) of 10.16 ± 1.69 µg/100 µL against the HepG2 cell line, which was comparable to 5-fluorouracil (standard, CC_50_ = 8.59 ± 1.33 µg/100 µL). In silico molecular docking suggested δ-curcumene, *Ar*-curcumene, *Ar*-turmerol, and *Ar*-turmerone among the promising compounds based on their high binding energy scores with NOX2, NF-κB, and mdm2 proteins. In conclusion, the oils from the turmeric–black cumin combined possess a considerable inhibition ability against free radicals, protein denaturation, and cancer proliferation. This study’s findings further underscore the effectiveness of turmeric–black cumin as a polyherbal medicinal ingredient.

## 1. Introduction

Reactive oxygen species (ROS) are chemical species that are formed upon incomplete reduction of oxygen in the biological system [1]. They are oxidants, which include singlet oxygen (1O_2_), hydroxyl radicals (·OH), hydrogen peroxide (H_2_O_2_), and superoxide anions (O_2_·^−^) [2]. They become highly reactive in nature and can kill cells in the body by oxidizing cellular components, including proteins, lipids, and nucleic acids, thus causing inflammation [3]. ROS affect multiple inflammatory signaling pathways, including the nod-like receptor family pyrin domain-containing 3 (NLRP3) inflammasome, the nuclear factor kappa B (NF-κB), the mitogen-activated protein kinase (MAPK), the Janus kinase-signal transducer and activator of transcription (JAK/STAT), the nuclear factor erythroid 2-related factor 2 (Nrf2), and the phosphatidylinositol 3-kinase and protein kinase B (PI3K/Akt) signaling pathways [4]. Their excessive production over endogenous antioxidant defense mechanisms contribute to the emergence of oxidative stress, which contributes to the etiology of several chronic inflammatory diseases such as cardiovascular and autoimmune diseases, diabetes, and cancer [5,6].

Antioxidants are a group of biologically active substances that protect cells and tissues of living organisms against the harmful effects of free radicals by ultimately inactivating ROS [7]. Studies have shown that antioxidants have the capacity to influence key signaling pathways responsible for inflammation. For instance, they can modulate the activity of NF-κB, MAPK, tumor necrosis factor (TNF-α) transcription, and arachidone pathways to reduce inflammation in cells [7,8]. Synthetic antioxidants such as butylated hydroxy anisole, butylated hydroxy toluene, and propyl gallate are associated with alterations in sleeping, inducing changes in brain serotonin and norepinephrine levels, thyroid system damage, metabolic and growth disorders, neurotoxicity, carcinogenesis, and allergic contact dermatitis [9,10,11]. Non-steroidal anti-inflammatory drugs (NSAIDs) such as diclofenac, ibuprofen, naproxen, nimesulide, and sulindac can cause dyspepsia, ulcers, gastro-intestinal bleeding, hypertension, and stroke in the cause of inflammation management [12]. In the same vein, significant incidences of hypokalemia, hypophosphatemia, mucosal inflammation, stomatitis, nephrotoxicity, neurotoxicity, and ototoxicity have been reported with the use of the anticancer drugs 5-fluorouracil and cisplatin [13,14]. Thus, there is a continuous search for less toxic and potent natural products that can effectively inhibit free radicals, inflammation, and cancer proliferation.

*Curcuma longa* L. (Zingiberaceae) and *Nigella sativa* L. (Ranunculaceae), commonly called turmeric and black cumin, respectively, are two essential oil-bearing (aromatic) plants, which are widely exploited for their medicinal values [15,16]. Reports have shown that the rhizomes of turmeric and black cumin seeds are part of the ingredients used in traditional medicine for the management of rheumatism, metabolic diseases, cancer, and other inflammatory ailments [17,18,19]. The potential health benefits of turmeric essential oils (EOs), as natural antioxidant, anti-inflammatory, and antinociceptive agents, have been reported with high composition of aromatic *Ar*-turmerone, curlone, and *Ar*-curcumene [20]. Similarly, the antioxidant, anti-inflammatory, and anticancer activities of black cumin oil have been documented, with thymoquinone, *p*-cymene, and α-phellandrene among its major constituents [21,22].

Turmeric rhizomes and black cumin seeds are often combined in Nigerian ethnomedicine for the management of cancer and other chronic inflammatory diseases [23]. A turmeric–black cumin polyherbal mixture from Pakistan has been reported to demonstrate enhanced efficacy against metabolic syndrome both in fructose-fed rats and in clinical studies [18,19]. Currently, there is a dearth of information on the antioxidant, anti-inflammatory, and anticancer properties of these spices in their combined form as well as their likely bioactive compounds. In recent times, in silico studies are mostly employed in the process of drug discovery from natural products (NPs), in conjunction with the acquisition of in vitro data, in order to build models that facilitate not only the biological potential of NPs but also the identification and refinement of lead compounds by providing insight into their druglike features in a timely and cost-effective manner [24,25]. It is on this backdrop that this study reported, for the first time, six batches of hydro-distilled EOs from a turmeric–black cumin herbal combination as well as their capacity to inhibit free radicals, protein denaturation, and cancer cell proliferation in vitro. Furthermore, the putative EO compounds were screened for their activity against three proteins implicated in ROS, inflammation, and cancer.

The ROS protein nicotinamide adenine dinucleotide phosphate (NADPH) oxidase 2 (NOX2) is a multi-subunit enzyme complex that participates in the generation of superoxide or hydrogen peroxide (H_2_O_2_). The hyperactivation of NOX2 increases oxidative stress, which is involved in the pathogenesis of several diseases [26]. The inflammatory protein NF-κβ is known to mediate cytokine production and cell viability and has occasionally been linked to the etiology of cancer and autoimmune diseases [27]. Lastly, the cancer protein E3 ubiquitin-protein ligase (mdm2), employed in this study, functions as a negative regulator of the tumor suppressor gene (p53), increasing the risk of cancer [28]. Therefore, sourcing natural products that can effectively inhibit the NOX2, NF-κβ, and mdm2 proteins may be a worthwhile strategy for the identification of ROS, inflammation, and cancer drug candidates, respectively. Thus, the EO compounds identified in the turmeric–black cumin combined, were further potentiated against the NOX2 (7U8G), NF-κβ (1NFK), and mdm2 (3W69) proteins following in silico molecular docking studies.

## 2. Results and Discussion

### 2.1. Physicochemical Characterization and TLC–Bioautography of Turmeric–Black Cumin Oils

**Figure 1:**
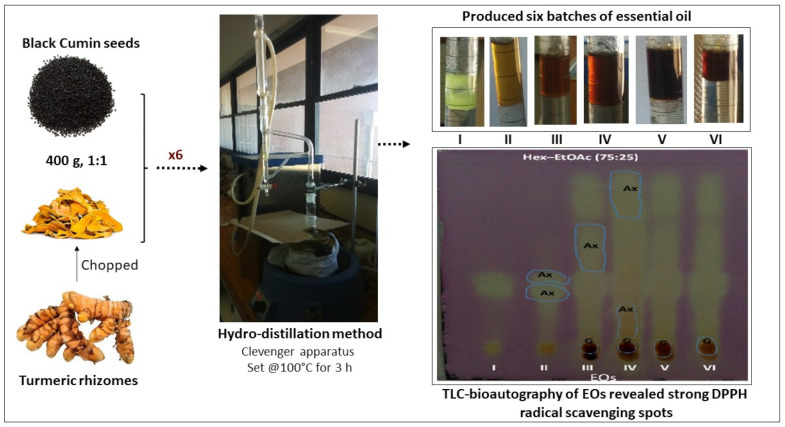
Six batches of essential oils from the hydro-distillation process, showing variation in oil color and free-radical-scavenging properties. [TLC-bioautography of the oils revealed yellowish spots against the purple DPPH background, indicating the presence of antioxidant (Ax) constituents in the oils].

Six batches of essential oils (EO I–VI) were obtained from the HD of Turmeric–black cumin combined, as presented in Table 1. Generally, the EOs gave a woody aroma. It is of note that the oils became roasty in aroma following the collection of batches III–V. The spice mixture later became smoky during the collection of batch VI and had a foamy appearance, indicating exhaustive HD of the oil from the herbal material. There was a marked increase in the oil yield upon repeated extraction from batches I–IV, followed by a noticeable reduction during extraction from batches IV–VI. As illustrated in Figure 1, there was an observable change in the color intensity of the oils, from yellow, through red, to a dark brown appearance. A preliminary assessment of the 2,2-diphenyl-1-picrylhydrazyl (DPPH) rapid radical action of the EOs based on TLC-bioautography (Figure 1) revealed the presence of strong free-radical-scavenging constituents, with batches III–VI having more of such antioxidant (Ax) constituents. It is possible that repeated HD may be considered for obtaining a good-quality yield of the free-radical-scavenging components from the Turmeric–black cumin combined.

### 2.2. Chemical Composition of Turmeric–Black Cumin Essential Oils

Gas chromatography–mass spectrometry (GC-MS) is a conventional hybrid method designed for the separation and characterization of volatile and non-volatile constituents of plant extracts. It is a specific, sensitive, linear, accurate, and precise analytical technique for analyzing multicomponent plant-based substances containing terpenes [29]. The results of the GC-MS analysis of the turmeric–black cumin EOs are presented in Table 2. A total of twenty-two (22) major constituents were identified in the six oil batches. Three sesquiterpenoids, *Ar*-turmerone, curlone, and α-turmerone, were among the major constituents in the oils. Also observed was a steady increase in the percentage composition of *Ar*-turmerone upon repeated HD from batches I–V, with contents of 17.17% in batch I, 54.88% in batch III, and 91.97% in batch V. Studies have shown that turmerones in their *Ar*, α, and β (curlone) forms are part of the major constituents of turmeric oil [30,31,32], while they have also been reported to be present in black cumin seeds in trace amounts [33]. The presence of *o*- and *p*-cymene, α-phellandrene, *o*-guaiacol, and fatty acids (palmitic, oleic, and stearic acids) in the oils were contributed by the black cumin seeds, as these constituents have been reported in this plant [34,35].

### 2.3. Free-Radical-Scavenging Activity

Spectrophotometric methods are analytical techniques employed for the quantitative measurement of the free-radical-scavenging activity of natural products due to their sensitivity, reproducibility, rapidness, and cost-effectiveness [39]. They measure the relative abilities of antioxidants in any particular extract to scavenge free radicals in comparison with standard antioxidants such as L-ascorbic acid, gallic acid, and trolox [39]. In this study, spectrophotometric methods such as the 2,2-diphenyl-1-picrylhydrazyl (DPPH), hydrogen peroxide (H_2_O_2_), nitric oxide (NO), and ferric ion (Fe^3+^) radical scavenging assay methods were used to determine the antioxidant activities of the EOs obtained from the turmeric–black cumin combined spice, as indicated by the 50% inhibitory concentration (IC_50_) of the radicals as well as the ascorbic acid equivalent (AAE).

The results (Table 3) showed a general improvement in the free-radical-scavenging capacity of the turmeric–black cumin herbal combination upon repeated HD, with the batch VI oil showing more activity than each of the separate oils. The batch VI oil, with *Ar*-turmerone (58.82%) and guaiacol (37.12%) as its major constituents, showed significantly (*p* < 0.05) better activity than the batch V oil containing mainly *Ar*-turmerone (91.97%), suggesting that both *Ar*-turmerone and o-guaiacol contributed to the antioxidant capacity of the former. Studies have shown that turmeric alone, having a 61.79% content of *Ar*-turmerone, is capable of scavenging free radicals such as DPPH, superoxide, and hydroxyl radicals at 1000, 135, and 200 µg/mL IC_50_ values, respectively [20]. Also, o-guaiacol, which was found to be the major component (39.12%) in black cumin, has previously been documented to exhibit strong antioxidant properties [40,41]. Therefore, it may not be far-fetched to mention that the repeated HD of the turmeric-black cumin combined afforded EOs with improved antioxidant activity, while *Ar*-turmerone and o-guaiacol may have jointly influenced the antioxidant activity of the oil in terms of scavenging free radicals. It is also worth mentioning that the remarkable free-radical-scavenging activity of the batch VI oil further underscores the usefulness of the two spices for polyherbal medicinal applications.

### 2.4. Inhibitory Effect of Oils on Protein Denaturation

**Figure 2:**
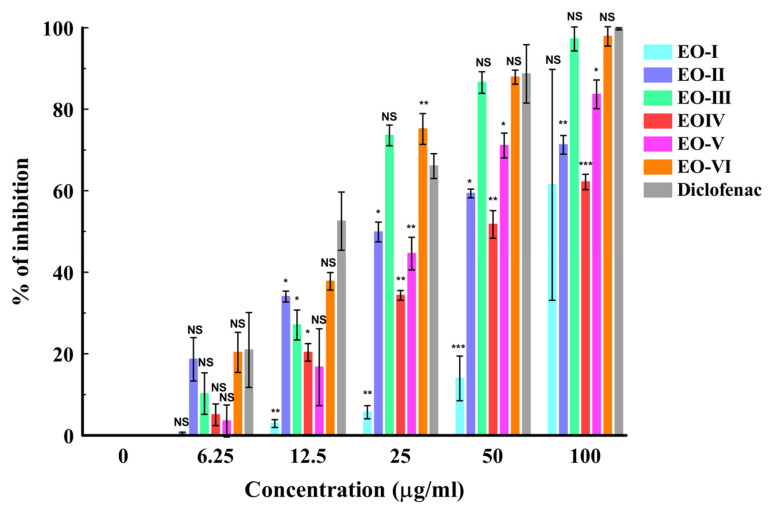
In vitro anti-inflammatory activity of essential oils from turmeric–black cumin combined. [Results are given as mean ± S.D. (*n* = 3). * *p* < 0.05; ** *p* < 0.01; *** *p* < 0.001; NS = non-significant, six batches of oil (EO-I–EO-VI)].

**Figure 3:**
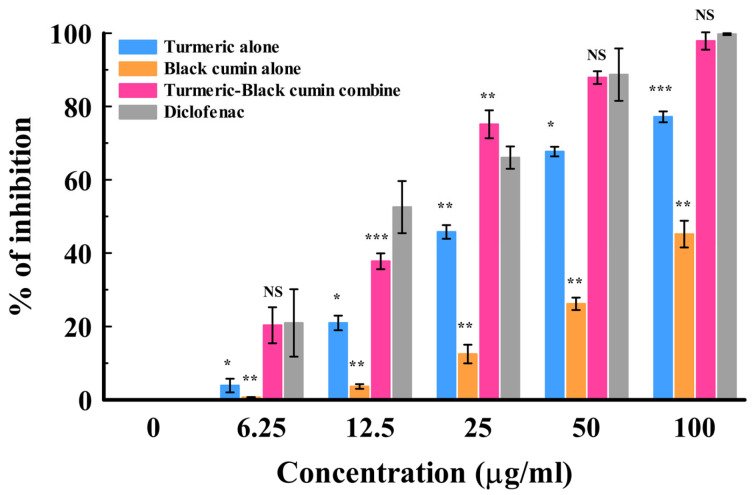
In vitro anti-inflammatory activity of essential oils from turmeric–black cumin combined in comparison with separate oils. [Results are given as mean ± S.D. (*n* = 3). * *p* < 0.05; ** *p* < 0.01; *** *p* < 0.001; NS = non-significant].

Protein denaturation has been well correlated with the occurrence of chronic inflammatory diseases including cancer [42,43]. The egg albumin (protein) denaturation test is a fast and reliable analytical technique for assessing the anti-inflammatory response of natural products [44]. The mean percentage inhibitions of the oils from batches I–VI on egg albumin denaturation are presented in Figure 2. The results showed a concentration-dependent increase in inhibition. At the lowest test concentration, diclofenac demonstrated comparable activity to the oils from batches VI, III, and II with about 20% inhibition. Moreover, at a higher concentration of 25, the batch VI oil exhibited a 75.15 ± 2.19% inhibition, which was significantly (*p* < 0.01) higher in activity compared to diclofenac (66.05 ± 1.77% inhibition) but comparable with the batch III oil (73.56 ± 1.47% inhibition). At 50 and 100 µg/mL, batches III and VI and diclofenac were all comparable. The 50% inhibitory effects of the oils (IC_50_) assisted in the final ranking of the activity of the EOs. Overall, the batch VI oil demonstrated the best activity, with an IC_50_ of 25.36 ± 1.61 µg/mL. Interestingly, the activity displayed by the batch VI oil was comparable with diclofenac, with an IC_50_ of 21.57 ± 2.66 µg/mL. The study findings are in agreement with the antioxidant activity pattern of the oils described earlier. This may imply that their inhibitory activity may be due to the displayed free-radical-scavenging activity. *Ar*-Turmerone and guaiacol, which were the major constituents identified in the batch VI oil, are known anti-inflammatory agents. *Ar*-Turmerone has been reported to alleviate skin inflammation in HaCaT keratinocytes by inactivating the Hedgehog pathway [45], while the latter has been reported to demonstrate anti-inflammatory activity by inhibiting lipopolysaccharide (LPS)-stimulated NF-κB activation and cyclooxygenase (COX)-2 gene expression in a murine macrophage cell line (RAW 264.7) [46]. Further assessment was carried out to determine the potency level of the oils as separate entities (turmeric alone and black cumin alone) and in combination (batch VI oil) (Figure 3). The batch VI oil showed a higher inhibition of EAD than each of the separate oils across the tested concentrations. At 25 µg/mL, it exhibited a significantly (*p* < 0.01) higher activity (76.82 ± 2.31% inhibition) than diclofenac (68.27 ± 1.53% inhibition) at the same concentration. However, it exhibited comparable activity with diclofenac at higher concentrations (50 and 100 µg/mL). The activity (IC_50_) ranking of the tested samples is presented as follows: diclofenac (17.19 ± 1.88 µg/mL) > batch VI oil (22.17 ± 2.19 µg/mL) > turmeric oil (46.33 ± 0.97 µg/mL) > black cumin oil (105.56 ± 3.37 µg/mL). This study has also shown that a combination of turmeric and black cumin may better attenuate protein denaturation, leading to an enhanced anti-inflammatory response than when utilized as separate herbal ingredients.

### 2.5. Anticancer Activity

The MTT cell viability test is a versatile and popular colorimetric assay method that involves the conversion of the water-soluble yellow dye MTT [3-(4,5-dimethylthiazol-2-yl)-2,5-diphenyltetrazolium bromide] to an insoluble purple formazan by the action of mitochondrial reductase [47]. The formazan is then solubilized, and the concentration is determined by the optical density, producing excellent linearity up to ~10^6^ cells per well and allowing one to detect cell stress upon exposure to cytotoxic agents [47]. The MTT assay method was used in this study to determine the level of viability of HEK293, HepG2, and HeLa cell lines after treatment with the turmeric–black cumin oils at varying concentrations and under standard conditions, thus evaluating the potency (cytotoxicity) of the oils, as presented in Table 4. The results showed that each of the six oil batches demonstrated a lower level of cytotoxicity to the non-tumorigenic cell line HEK293 compared to 5-fluorouracil, which gave a CC_50_ value of 16.18 ± 1.43 µg/100 µL. However, the black cumin oil alone showed the highest cytotoxicity to this cell line among the oils at a CC_50_ value of 37.16 ± 2.17 µg/mL. A previous chronic toxicity investigation of black cumin oil showed a mild toxicity at 2 mL/kg [48].

Further assessment of the cytotoxicity of the oils against the cancerous HeLa cell line showed batch VI, which contained *Ar*-turmerone and o-guaiacol, exhibiting the best activity among the oils. This was closely followed by the batch I oil (CC_50_ = 17.53 ± 1.11 µg/100 µL), which contained 18 of the 22 identified constituents. Additionally, the batch VI oil gave the best activity against the liver cancer cell line HepG2, with a CC_50_ of 10.16 ± 1.69 μg/100 µL, and this was comparable to 5-fluorouracil (CC_50_ = 8.59 ± 1.33 µg/100 µL) and the batch I oil (CC_50_ = 12.77 ± 2.38 µg/100 µL). Turmeric oil alone has been reported to exhibit 100% cytotoxicity against the pancreatic cancer cell line PANC-1 at 110.5 μg/mL, yielding a CC_50_ of 73.7 μg/mL for the unpurified oil fraction, and a CC_50_ of 23.3 μg/mL for the purified oil fraction [49]. Black cumin oil has also been reported to demonstrate a CC_50_ ≤ 250 µg/mL against the human liver cancer cell line A59 [50]. Based on the study findings, the oils from batches VI and I may be candidate cytotoxic agents against cervical (HeLa) and liver (HepG2) cancer cells. It has also been demonstrated through this study that turmeric and black cumin spices may be more effective as a combination rather than as separate administrations, while the improvement in the anticancer activity following repeated HD may be an indication to adopt this extraction method to obtain the putative bioactive constituents of the turmeric–black cumin combined.

### 2.6. In Silico Molecular Docking

**Figure 4:**
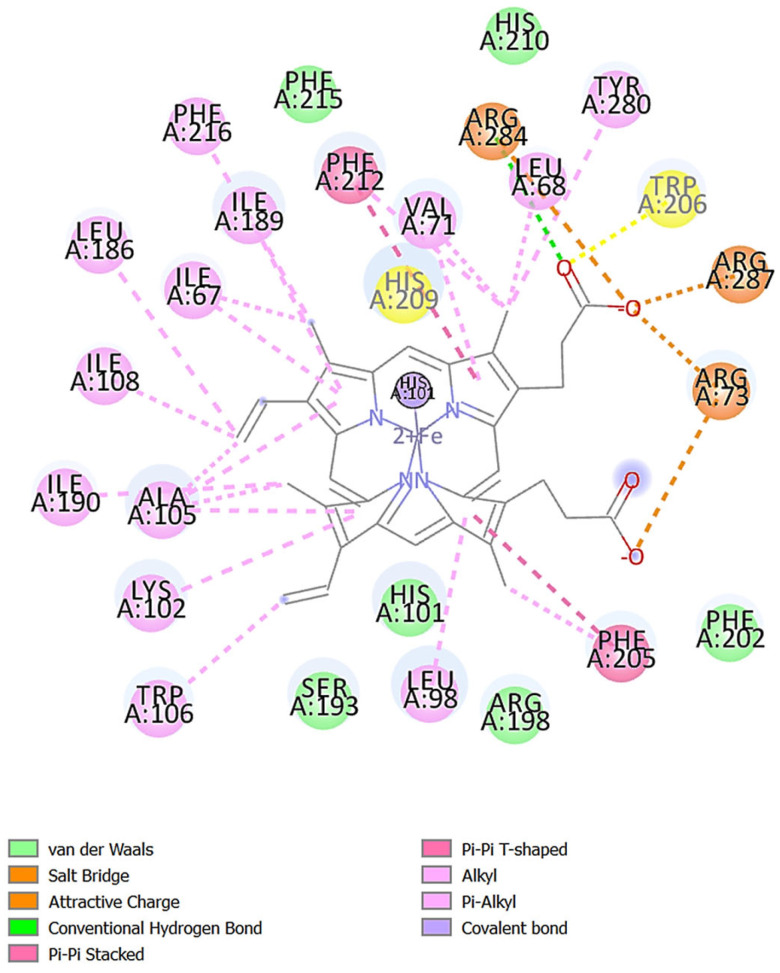
Amino acids formed the active site pocket in NOX2.

**Figure 5:**
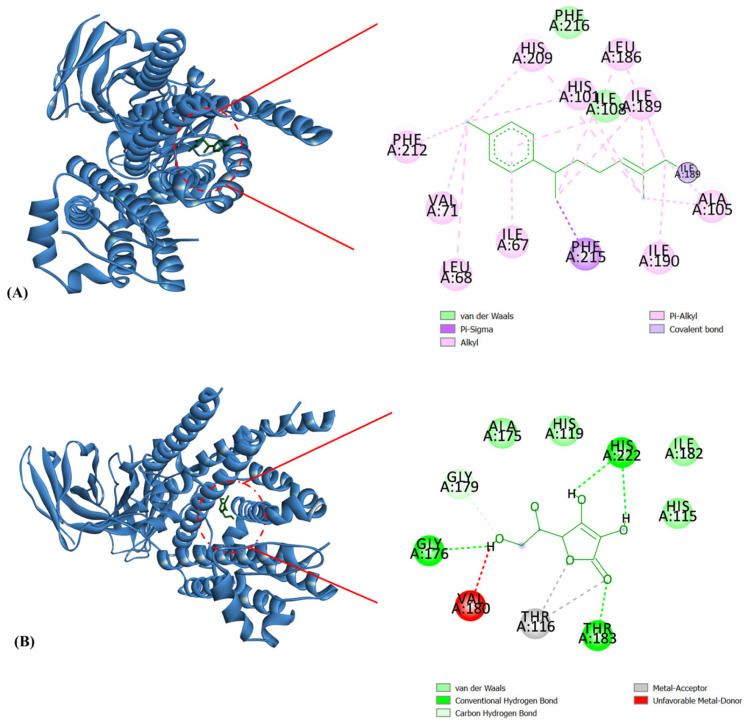
Molecular docking interactions of NOX2 with (**A**) *Ar*-curcumene and (**B**) L-ascorbic acid. The blue ribbon represents the NOX2 protein. *Ar*-Curcumene and L-ascorbic acid are illustrated as a green stick.

The major constituents identified in the EOs of the turmeric–black cumin combined were molecularly docked against the NOX2, NF-κβ, and mdm2 proteins. Table 5 displays the binding energy scores of the oil constituents against the NOX2 protein. The results of the study showed that Ile67, Leu186, Ile189, Phe216, Phe215, Phe212, His209, His101, Leu68, Val71, Arg284, His210, Tyr280, Trp206, Arg287, Arg73, Phe202, Phe205, Arg198, Leu98, His101, Ser193, Trp106, Lys102, Ala105, Ile190, and Ile108 formed the active site pocket in NOX2 (PDB ID 7U8G) (Figure 4). Among the twenty-two phytocompounds, *Ar*-curcumene showed the best binding energy score of −8.0 kcal/mol with hydrophobic interactions (Ile108, Phe216), Pi-alkyl interactions, including Ile67, Leu68, Val71, His 101, Ala105, Leu186, Ile189, Ile190, His209, and Phe212, and Pi-sigma interactions (Phe215) (Figure 5A). It is interesting to note that these residues found in the active site (Figure 4) were linked to *Ar*-curcumene, a substance that has been demonstrated to play a crucial part in the interaction between NOX2 and small-molecule inhibitors (Figure 5A). The enzyme NOX is a membrane-bound ROS producer. When the enzyme NOX moves an electron from one oxygen atom to another, superoxide is created. Catalase (CAT) uses reduced glutathione (GSH) to further catalyze the conversion of H_2_O_2_, during which it first converted from superoxide to water [51]. The standard L-ascorbic acid showed a binding energy score of −5.7 kcal/mol with hydrophobic interactions (His115, His119, Ala175, and Ile182) and hydrogen bond interactions (Gly176, Gly179, Thr183, and His222) (Table 5 and Figure 5B).

**Figure 6:**
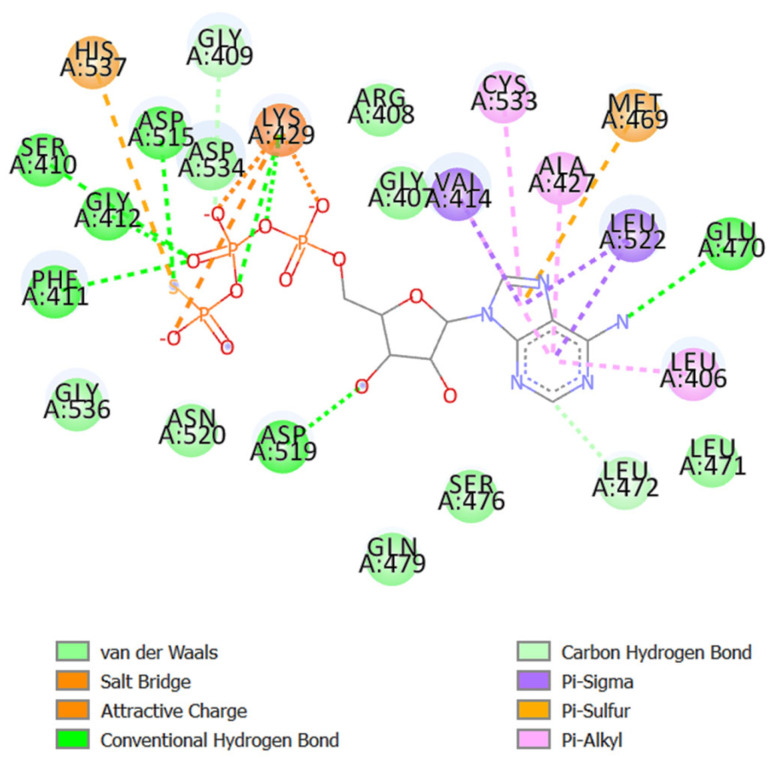
Amino acids formed the active site pocket in NF-κβ.

**Figure 7:**
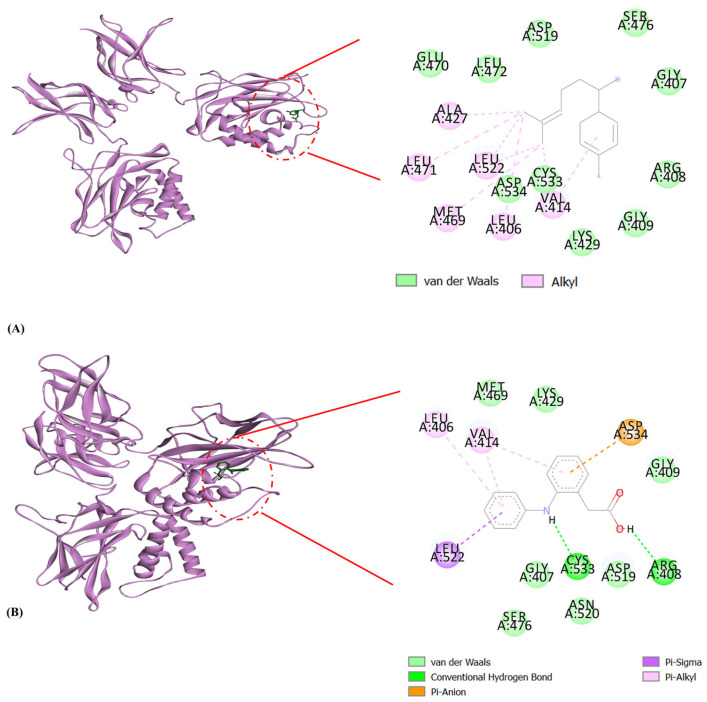
Molecular docking interactions of NFκβ with (**A**) δ-curcumene and (**B**) diclofenac. The pink ribbon represents the NFκβ protein. δ-Curcumene and diclofenac are illustrated as a green stick.

The NF-κβ protein and the native ligand produced the active site pocket (Figure 6). The molecule δ-curcumene was found to have a slight upper hand compared to the remaining constituents, returning a binding energy score of −8.1 kcal/mol with hydrophobic interactions (Arg161, Gly162, Asn164, Gly166, Leu176, Gln177, and Thr226) and Pi-alkyl interactions (Pro165, Leu167, Phe217, and Arg228) with the protein NF-κβ (Figure 7A). However, the standard diclofenac was not far behind, as it showed a value of −5.9 kcal/mol, with hydrophobic interactions (Gly162, Asn164, Leu173, Ala174, Gln177, Phe217, and Thr226), hydrogen bonds (Leu167), Pi-alkyl interactions (Pro165), and Pi-cation (Arg228) interactions (Table 5 and Figure 7B). Interestingly, Figure 6 shows that these active site residues were associated with δ-curcumene, a substance that has been shown to have a significant role in the interaction between mdm2 and small-molecule inhibitors (Figure 7A). NF-κβ is known to be a primary source of inflammation. It also regulates cell viability and cytokine production and has occasionally been linked to autoimmune disorders and cancer [52]. Therefore, for illnesses associated with inflammation, blocking this protein may be a good strategy.

**Figure 8:**
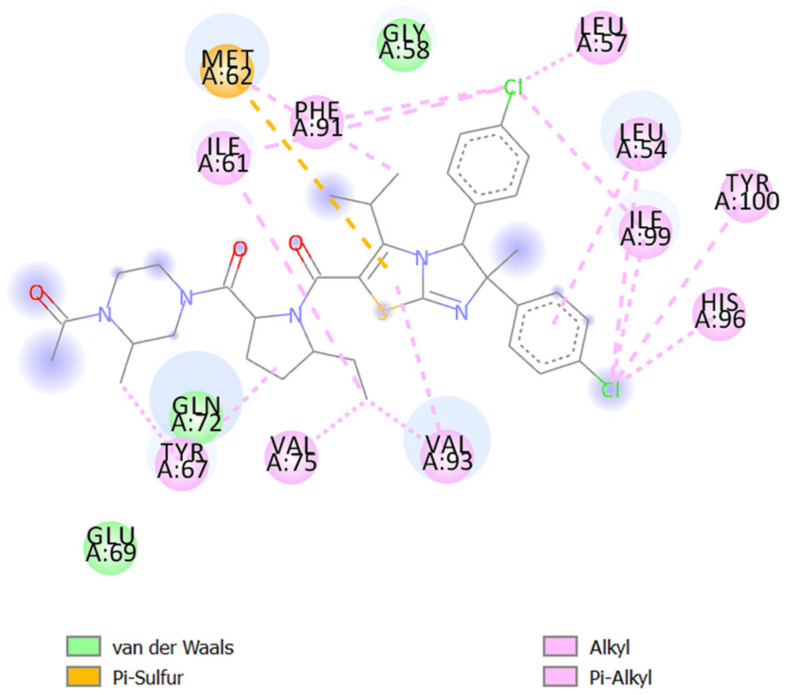
Amino acids formed the active site pocket in mdm2.

**Figure 9:**
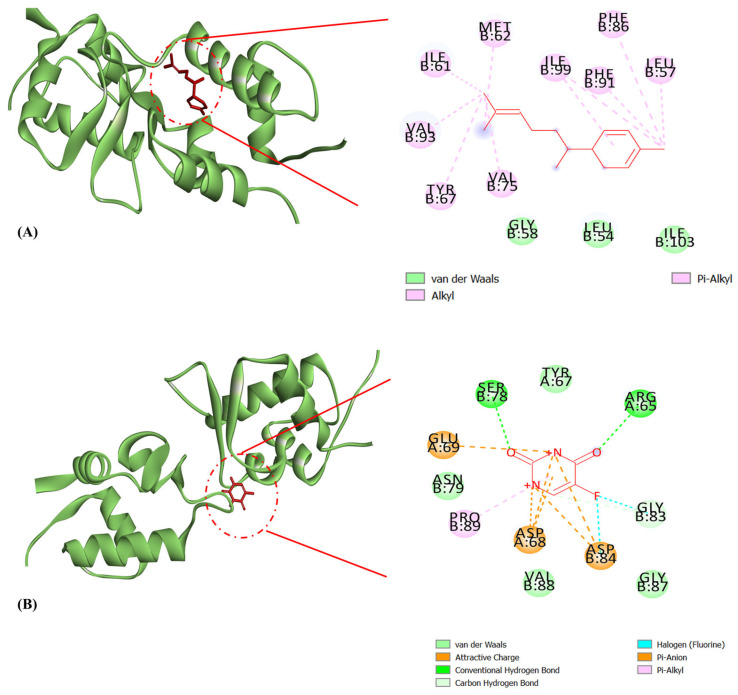
Molecular docking interactions of mdm2 with (**A**) δ-curcumene and (**B**) 5-fluorouracil. The green ribbon represents the mdm2 protein. δ-Curcumene and 5-fluorouracil are illustrated as a red stick.

The active site residues in mdm2 are depicted in Figure 8. The active regions in the tertiary structures of enzymes are often located in a “cleft”, which requires substrate and product diffusion. Due to the folding necessary for the tertiary structure, the amino acid residues of the active site may be separated greatly in the primary structure [53]. Changes in the structure of amino acids at or near the active site of an enzyme typically affect the activity of the enzyme because of their important involvement in substrate binding and attraction. Proteins can frequently be stabilized through complexation with small molecules, nucleic acids, substrates, or cofactors [53]. A binding energy score of −8.5 kcal/mol was observed in the interaction of δ-curcumene with hydrophobic interactions (Leu54, Gly58, and Ile103) and Pi-alkyl interactions (Leu57, Ile61, Met62, Tyr67, Val75, Phe86, Phe91, Val93, and Ile99) with the mdm2 protein (Table 5 and Figure 9A). The standard 5-fluorouracil showed a binding energy score of −4.7 kcal/mol with hydrophobic interactions (Tyr67, Gly87, Asn79, and Val88), hydrogen bonding (Arg65, Ser78, and Gly83), Pi-alkyl interactions (Pro89) and Pi-anion interactions (Asp68, Glu69, and Asp84) (Table 5 and Figure 9B). Interestingly, these residues identified in the active site (Figure 8) were associated with δ-curcumene, which is essential for the interaction of mdm2 with small-molecule inhibitors (Figure 9A). It is also worth mentioning that 5-fluorouracil did not bind to any of the active site residues of the mdm2 protein, unlike δ-curcumene. This in silico result might be the reason for the lower binding energy score of 5-fluorouracil (−4.7 kcal/mol) compared to δ-curcumene (−8.5 kcal/mol). Thus, δ-curcumene showed considerable outcomes in terms of its in silico analysis. Studies have shown the involvement of α-curcumene from *Curcuma zedoara* in the in vitro anti-proliferation of the ovarian cancer cell line SiHa based on the concentration-dependent activation of caspase-3, which is one of the main executors of the apoptotic process [54].

## 3. Materials and Methods

### 3.1. Preparation of Herbal Material

The rhizomes of *Curcuma longa* (turmeric) and the seeds of *Nigella sativa* (black cumin) were collected from Osogbo, Osun State, Nigeria (latitude 7°33′00″ N and longitude 4°33′00″ E). They were authenticated by a plant taxonomist, Herbert C. Illoh, while the herbarium specimens were deposited at the Ife Herbarium, Obafemi Awolowo University (Ile-Ife, Nigeria), with voucher numbers 18,597 (turmeric) and 18,599 (black cumin) for future reference.

### 3.2. Hydro-Distillation (HD) of Turmeric–Black Cumin Combined Spice

A total of 200 g each of the chopped rhizomes of turmeric and the seeds of black cumin were mixed and introduced into a 5 L round-bottomed flask containing 2.5 L of distilled water. The combined spice was hydro-distilled for its EOs on a Clevenger apparatus, as described by Oyedeji et al. [55] with slight modification. The heating mantle temperature was set at 100 °C with constant boiling for 3 h, after which it was allowed to cool. Then, the first batch of oil was collected over n-hexane, followed by drying over anhydrous sodium sulfate to remove excess solvent from the oil. The HD was repeated five times until there was no visible sign of oil distilling out of the system. The afforded batches of oil were collected in amber vials and refrigerated until further analysis.

### 3.3. Physicochemical Analyses of Turmeric–Black Cumin Oils

The EOs were physically characterized by color, odor, and percentage yield. Thin-layer chromatography (TLC)-bioautography was carried out on Silica gel 60 F254 GF plates (0.25 mm, Merck KGaA, Darmstadt, Germany), with the TLC plates developed in a pre-saturated tank of n-hexane–ethyl acetate (9:1) in duplicate. The chromatograms were sprayed with 10% sulfuric acid and 2,2-diphenyl-1-picrylhydrazyl (DPPH) radical (Sigma-Aldrich, St. Louis, MO, USA) for the general detection of organic compounds and the qualitative assessment of the free-radical-scavenging properties, respectively [56].

### 3.4. GC-MS Analysis of Turmeric–Black Cumin Oils

The EOs were chemically analyzed on a Bruker 450 Gas Chromatograph connected to a 300 MS/MS mass spectrometer system (Karlsruhe, Germany) following the method of Miya et al. [57]. The GC-MS system was operated in an electrospray ionization (EI) mode at 70 eV. The gas chromatogram comprised an HP-5 MS fused silica capillary system (Agilent, Santa Clara, CA, USA) with 5% phenylmethylsiloxane as the stationary phase. The capillary column parameters were 30 m in length by 0.25 mm in internal diameter by 0.25 μm in film thickness. The column temperature was increased from 50–240 °C at a rate of 5 °C/min, while the final temperature was maintained at 450 °C for a duration of 66 min. The carrier gas was helium (1.0 mL/min flow rate), the scanning range was 35–450 amu, and the split ratio was 100:1. One microliter (1 μL) of the diluted oil (50 µL oil:350 µL hexane) was injected for the analysis. The percentage composition of the chemical constituents was computed from the GC peak areas. Identification of the oil constituents was conducted based on the spectral matching of their fragmentation patterns, with the constituents recorded in the system database. Each constituent was confirmed by comparison of its Kovats’ index (KI) to those reported in the NIST Standard Reference Database and other sources in the literature [36,37,38].

### 3.5. Free Radical Scavenging Analyses of EOs

#### 3.5.1. 2,2-Diphenyl-1-picrylhydrazyl (DPPH) Spectrophotometric Assay

This was carried out by following the standard experimental procedures for DPPH assays [58]. An amount (0.5 mL) of 0.1 mM DPPH radical in methanol was added to 0.5 mL of serially diluted test samples (EOs and L-ascorbic acid) at 100, 50, 25, 12.5, 6.25, and 3.125 µg/mL concentrations in triplicate. The separate oils of turmeric and black cumin were also tested for reference purposes. The reaction mixture was incubated in the dark at 25 °C for 30 min. The absorbance was measured at 517 nm on a 680-Bio-Rad Microplate Reader (Serial Number 14966, Hercules, CA, USA). The percentage inhibition of the radical was calculated according to Equation (1):

(1) $$\%\;Inhibition=\frac{ABScontrol-ABSsample}{ABScontrol}\times 100$$

where *ABSsample* = absorbance of the test sample, i.e., EOs and L-ascorbic acid. *ABScontrol* = absorbance of the negative control (methanol).

#### 3.5.2. Nitric Oxide (NO) Inhibition Assay

The inhibitory effect of the EOs against the NO radical was investigated using the method described by Jimoh et al. [59]. Here, 0.5 mL of the test samples at varying concentrations (100–3.125 µg/mL) was added to sodium nitroprusside (2 mL, 0.2 mM) in triplicate. The reaction mixture was incubated at 25 °C for 3 h. Then, 0.5 mL of the mixture was mixed with Griess reagent [0.33% sulphanilamide dissolved in 20% glacial acetic acid and mixed with 1 mL of naphthylethylenediamine chloride (0.1% *w*/*v*)]. The mixture of the complex and Griess reagent was then incubated at room temperature for 30 min. Thereafter, it was measured at an absorbance of 540 nm on a 680-Bio-Rad Microplate Reader (Serial Number 14966, Hercules, CA, USA). The percentage inhibition of the NO radical was calculated using Equation (1).

#### 3.5.3. Hydrogen Peroxide Inhibition Assay

The ability of the essential oils to inhibit the H_2_O_2_ radical was measured using the standard colorimetric method, as described by Okeleye et al. [60]. The test samples (400 µL each) were serially diluted from 100 to 3.125 µg/mL concentrations and mixed with 60 µL of hydrogen peroxide solution (4 mM) prepared in 0.1 M phosphate-buffered saline (pH 7.4) in triplicate inside a 96-well plate. The reaction mixture was incubated at room temperature (≈25 °C) for 10 min. Thereafter, the absorbance was measured at 405 nm on a 680-Bio-Rad Microplate Reader (Serial Number 14966, Hercules, CA, USA). The percentage inhibition of the peroxyl radical was determined using Equation (1).

#### 3.5.4. Ferric-Reducing Antioxidant Power (FRAP) Assay

This procedure was conducted based on the ability of the EOs to reduce the greenish ferric ion (Fe^3+^) 2,4,6-tri-(2-pyridyl)-1,3,5-triazine (TPTZ) to the bluish ferrous ion (Fe^2+^) at a 593 nm absorbance measurement, as previously described by Benzie and Strain [61]. Thus, the ferric-reducing power of the essential oils was determined as the ascorbic acid equivalent (AAE) from the calibration curve of the positive control (L-ascorbic acid) at concentrations of 1000, 500, 250, 125, 62.5, and 31.25 µg/mL in methanol.

### 3.6. In Vitro Anti-Inflammatory Analysis of EOs

This procedure was performed using the protein denaturation assay method described by Chatterjee et al. [62]. A reaction mixture comprising 0.2 mL of the albumin content of fresh chicken egg, 2.8 mL of phosphate-buffered saline (pH 6.4), and 2 mL each of the EOs at varying concentrations (100–6.25 µg/mL) was prepared in triplicate. The mixture was incubated at 37 °C for 15 min away from direct light and thereafter boiled at 70 °C for 5 min in a thermostatic water bath. The resulting mixture was cooled, and the absorbance was measured at 655 nm on a 680-Bio-Rad Microplate Reader (Serial Number 14966, Hercules, CA, USA). Diclofenac and separate oils of turmeric and black cumin were also tested for reference purposes. The percentage inhibition of the test samples was calculated according to Equation (1).

### 3.7. Cytotoxicity Study

#### 3.7.1. Cell Culture

The human hepatocarcinoma (HepG2) and cervical (HeLa) cancer cell lines were supplied by Highveld Biologicals (Pty) Ltd., Lyndhurst, South Africa. The non-tumorigenic HEK293 cell line, which served as the control, was obtained from the University of Witwatersrand Medical School, South Africa. The culture medium comprised Eagle’s Minimal Essential Medium (EMEM) with L-glutamine from Lonza BioWhittaker (Verviers, Belgium), fetal bovine serum (FBS) from HyClone UK Ltd. (Cramlington, Northumberland, UK), and penicillin/streptomycin mixture (10,000 U/mL penicillin, 10,000 μg/mL streptomycin) from Lonza BioWhittaker (Verviers, Belgium). Sterile plastic wares for the cell culture were purchased from Corning Inc. (Corning, NY, USA), while 3-(4,5-dimethylthiazol-2-yl)-2,5-diphenyltetrazolium bromide (MTT) salt and dimethylsulphoxide (DMSO) were purchased from Merck, Darmstadt, Germany.

#### 3.7.2. MTT Assay

The cytotoxicity of the EOs against the HEK293, HepG2, and HeLa cell lines was determined based on the MTT assay method that was previously described by Jagaran and Singh [63]. The three cell lines were propagated in growth medium (EMEM supplemented with 10% (*v*/*v*) FBS, 100 U/mL penicillin, and 100 μg/mL streptomycin). Cells were seeded at an average density of 25,000 cells/well in 96-well plates and maintained at 37 °C for 24 h to reach semi-confluency. The cells were prepared by draining the wells and adding fresh medium (100 μL/well). The EOs were dissolved in 10% (*v*/*v*) DMSO with brief vortexing and sonication. Stock concentrations of the oils comprising 1, 2.5, 5, 7.5, and 10 μg/μL were prepared. The test samples, which comprised the combined spice oils (10 μL each), the standard anticancer drug (5-fluorouracil), and the reference oils (turmeric alone and black cumin alone), were introduced to give final concentrations of 10, 25, 50, 75, and 100 μg/100 µL in triplicate. The final concentration of DMSO to which the treated cells were exposed was 1% (*v*/*v*). Untreated cells were included as positive (100% cell viability) controls. Cells treated with 10% (*v*/*v*) DMSO (10 μL/well) served as additional controls. Cells were incubated at 37 °C for 48 h. The growth medium was aspirated, and the cells were incubated (37 °C, 4 h) with 100 μL each of the medium and MTT solution (5 mg/mL in PBS) per well. The wells were drained, and formazan crystals were dissolved in DMSO (100 μL/well) to give purple-colored solutions. Absorbance was read at 540 nm in a Mindray microplate reader, MR 96A (Vacutec, Hamburg, Germany), against pure DMSO as a blank. The percentage cell viability was calculated as per Equation (2):

(2) $$\frac{\left[A_{540nm}(treatedcells)-A_{540nm}(blank)\right]}{\left[A_{540nm}(untreatedcells)-A_{540nm}(blank)\right]}\times 100$$

The concentration that showed 50% cytotoxicity (reduced the viability of each cell line by 50%) was determined as the CC_50_ value of each test sample in µg/100 µL.

### 3.8. In Silico Molecular Docking of EO Constituents

#### 3.8.1. Preparation and Refinement of the Protein and Ligand Structures

The investigation involving molecular docking was carried out using the major compounds identified in the EOs of turmeric–black cumin combined against oxidative stress and inflammation. The PDB structures of NOX 2, NF-κβ, and mdm2, with PDB Ids 7U8G [64], 1NFK [65] and 3W69 [66], respectively, were acquired from the Protein Data Bank (http://www.rcsb.org). To prepare the protein structures for docking, polar hydrogen atoms and Kollman charges were added after the removal of water atoms using AutoDockTools. The concerned phytocompounds were downloaded from NCBI PubChem (https://pubchem.ncbi.nlm.nih.gov/, accessed on 12 May 2024). The Open Babel Server was then used to transform the downloaded sdf structures into pdb structures [67]. The ligand structures underwent energy minimization using a Gromos 96 force field after the PRODRG server [68] was used to optimize their energy.

#### 3.8.2. Determination of the Active Site and Molecular Docking

The active sites of the proteins (NOX2, NF-κβ, and mdm2) were predicted using the literature [64,66,69] and validated through the CASTp 3.0 (Computed Atlas of Surface Topography of Proteins) online server [70]. The processed proteins without a native inhibitor were uploaded to the CASTp 3.0 server, and the top result from the best 3 potential ligand-binding sites was chosen for docking. The amino acid residues predicted by CASTp 3.0 were then compared with the amino acids in the active site of the native inhibitor–NOX2 co-crystallized complex, native inhibitor–NFκβ co-crystallized complex, and native inhibitor–mdm2 co-crystallized complex. This was carried out by manually opening the co-crystallized complex in the Discovery studio visualization tool [71] to verify the active site. This allowed for the identification of the interacting residues, which were found to be quite similar to those predicted by the CASTp 3.0 server. The Autodock and Autogrid tools integrated with Autodock4 were used to generate grid maps (X, Y, and Z confirmations; Box center and Box dimension) for each atom of the native inhibitor ligand. In order to obtain the X, Y, and Z confirmations (Box center and Box dimension) as the potential target site, molecular docking was carried out using Autodock4. The ligands of interest were molecularly docked at the active binding sites of the relevant proteins utilizing a stiff protein receptor and a flexible ligand docking methodology through the use of a grid-based molecular docking technology [71,72]. A grid box involving the active site residues of the NOX2 protein was created, with center_x = 144.171, center_y = 141.09, center_z = 151.26, size_x = 21.0, size_y = 34.0, and size_z = 35.0. For, NFκβ protein with center_x = −8.0, center_y = 30.1, center_z = −4.6, size_x = 24.0, size_y = 15.1, and size_z = 18.9. Similarly, a grid box covering the active binding pocket of mdm2 was employed, with center_x = −34.4, center_y = 29.1, center_z = −11.1, size_x = 15.7, size_y = 18.5, and size_z = 28.9. After Autodock Vina finished the molecular docking procedure, the docked complexes were visualized using the Discovery Studio visualization tool [73].

### 3.9. Statistical Analysis

The in vitro antioxidant, anti-inflammatory, and cytotoxicity data were expressed as mean ± standard deviation (S.D.) using Microsoft Excel version 365 (Microsoft Corporation, Washington, DC, USA). The results were analyzed using one-way analysis of variance (ANOVA) followed by Student’s and Newman–Keuls’ post hoc tests on GraphPad Prism 5 (GraphPad Software Inc., San Diego, CA, USA).

## 4. Conclusions

This study presented six essential oil batches, I–VI, from turmeric–black cumin combined spice. Twenty-two (22) major constituents were identified in the oils, with *Ar*-turmerone being the most prominent compound. The oils, notably those of batch VI, inhibited DPPH, NO, H_2_O_2_, and Fe^3+^ free radical free radicals in vitro. They also attenuated the protein denaturation and proliferation of liver (HepG2) and cervical (HeLa) cancer cells. The in silico studies suggested δ-curcumene, *Ar*-curcumene, *Ar*-turmerol, and *Ar*-turmerone as the most promising compounds in the oils based on their high binding energy scores with the NOX2, NF-κβ, and mdm2 proteins implicated in ROS, inflammation, and cancer, respectively. Thus, the oils of a combination turmeric–black cumin possess a considerable inhibition ability against free radicals, protein denaturation, and cancer proliferation. This study’s findings further underscore the effectiveness of turmeric–black cumin as a polyherbal medicinal ingredient. Future research endeavors may examine the in vivo bioavailability, bioactivity, and safety profiles of the turmeric–black cumin essential oil combination and its putative compounds, notably δ-curcumene.

## Figures and Tables

**Table 1:** Physical properties of EOs from turmeric–black cumin combined.

Essential Oil	Color	Sensory Characteristics	% Yield
I	Yellow	Earthy, woody	0.35
II	Golden yellow	Earthy, woody	0.39
III	Red	Earthy, woody, roasty	0.41
IV	Reddish brown	Earthy, woody, nutty, roasty	0.43
V	Reddish brown	Woody, nutty, roasty	0.33
VI	Dark brown	Woody, nutty, smoky	0.25

Six batches of essential oils (I–VI), % yield expressed as *w*/*w* (g) × 100, starting weight of herbal material = 400 g.

**Table 2:** Chemical composition of essential oils from turmeric–black cumin combined.

S/N	Major Essential Oil Constituent	t_R_ (min)	KI ^a^	KI [36,37,38]	% Composition of Oil					
					I	II	III	IV	V	VI
1	α-Phellandrene	11.59	1004	1003	-	-	1.17	-	-	-
2	*p*-Cymene	12.35	1022	1020	4.38	-	-	-	-	-
3	*o*-Cymene	12.40	1025	1022	2.29	1.13	-	-	-	-
4	*p*-Cresol	12.51	1074	1071	-	-	13.28	-	-	-
5	*o*-Guaiacol	12.60	1090	1087	-	-	-	-	-	37.12
6	*Trans*-4-methoxy thujane	15.68	1124	1120	0.99	-	-	-	-	-
7	4-Methyl-1-(1-methylethyl)-3-cyclohexenol	17.58	1176	1171	2.26	-	-	-	-	-
8	*p*-Thymol	21.27	1334	1332	1.27	-	-	-	-	-
9	2-*epi*-α-Funebrene	22.63	1385	1380	0.82	-	-	-	-	-
10	*Ar*-Curcumene	26.33	1481	1480	4.01	-	-	-	-	-
11	δ-Curcumene	26.57	1483	1486	1.04	-	-	-	-	-
12	β-Sesquiphellandrene	27.03	1523	1521	2.43	-	-	-	-	-
13	*Ar*-Turmerol	28.34	1580	1582	-	1.85	-	-	-	-
14	*Ar*-Turmerone	30.39	1673	1672	17.17	46.39	54.88	59.61	91.97	58.82
15	α-Turmerone	31.15	1683	1680	9.21	18.22	7.13	-	-	-
16	Curlone	31.19	1701	1701	8.53	22.29	17.37	31.31	-	-
17	(*Z*)-γ-Atlantone	31.24	1744	1706	0.50	-	-	-	-	-
18	(*Z*)-α-Atlantone	31.42	1720	1717	0.53	0.50	-	-	-	-
19	(*E*)-α-Atlantone	32.70	1777	1773	2.18	2.51	2.46	-	-	-
20	Palmitic acid	36.86	1969	1965	29.21	-	-	-	-	-
21	Oleic acid	39.83	2142	2140	7.79	-	-	-	-	-
22	Stearic acid	40.17	2180	2177	1.43	-	-	-	-	-
	Total				96.04	92.89	96.29	90.92	91.97	95.94

^a^ Kovats’ index (KI) on HP-5 MS column fused with 5% phenylmethylsiloxane at 5 °C/min for 50–240 °C in reference to *n*-alkanes, absent (-), retention time (t_R_), six essential oil batches (I–VI).

**Table 3:** Antioxidant activity of essential oils from turmeric–black cumin combined.

Essential Oil	IC_50_ ± S.D. (µg/mL)			FRAP (mgAAE/g ± S.D.)
	DPPH	H_2_O_2_	NO	
I	63.29 ± 2.08	75.88 ± 4.15	41.62 ± 3.01	331.72 ± 23.79
II	30.41 ± 1.64	55.70 ± 2.72	49.83 ± 2.14	476.08 ± 34.04
III	33.15 ± 3.06	26.91 ± 2.16	20.49 ± 1.48	519.67 ± 18.25
IV	44.92 ± 2.55	30.24 ± 1.86	21.71 ± 2.62 *	488.63 ± 27.12
V	35.47 ± 2.21	32.41 ± 2.30	28.30 ± 2.55	451.85 ± 20.93
VI	14.14 ± 1.63 *	12.69 ± 1.55 *	22.36 ± 1.10 *	679.20 ± 31.37 **
Turmeric oil	30.19 ± 2.44	28.64 ± 1.75	26.79 ± 1.44	535.10 ± 12.33
Black cumin oil	61.58 ± 2.13	41.53 ± 2.62	43.56 ± 3.30	288.68 ± 17.05
L-ascorbic acid	8.91 ± 1.07 *	9.01 ± 0.81 *	11.49 ± 1.08 *	NA

Turmeric–black cumin essential oil batches (I–VI), data are expressed as mean ± standard deviation (S.D., *n* = 3), NA—not applicable, values significantly (*p* < 0.05) lower than turmeric oil are indicated with an asterisk (*), while samples with a double asterisk (**) indicate that the ferric-reducing antioxidant power (FRAP) of sample was higher than that of turmeric oil alone at *p* < 0.01.

**Table 4:** Anticancer activity of essential oils from turmeric–black cumin combined.

Essential Oil	CC_50_ ± S.D. (µg/100 µL)		
	HEK293	HepG2	HeLa
I	38.96 ± 2.02	12.77 ± 2.38 ^b^	17.53 ± 1.11 *
II	58.84 ± 3.18	26.30 ± 1.88 ^f^	28.92 ± 2.68
III	41.42 ± 2.62	32.18 ± 2.09 ^g^	39.74 ± 3.13
IV	76.60 ± 3.55	31.05 ± 2.61 ^g^	25.07 ± 2.06
V	47.06 ± 2.41	18.72 ± 1.17 ^c^	23.51 ± 2.12
VI	46.98 ± 1.80	10.16 ± 1.69 ^ab^	14.83 ± 2.05 *
Turmeric oil	42.97 ± 2.26	21.79 ± 1.01 ^d^	22.07 ± 2.22
Black cumin oil	37.16 ± 2.17 *	24.43 ± 1.64 ^e^	29.48 ± 2.57
5-Fluorouracil	16.18 ± 1.43 *	8.59 ± 1.33 ^a^	9.71 ± 1.25 *

Turmeric–black cumin essential oil batches (I–VI), non-tumorigenic cell line (HEK293), liver cancer cell line (HepG2), cervical cancer cell line (HeLa), data are expressed as mean ± standard deviation (S.D., *n* = 3), values significantly (*p* < 0.05) lower than turmeric oil alone are indicated with an asterisk (*), values with different alphabets in superscript were significantly different at *p* < 0.001, concentration that causes 50% cytotoxicity (CC_50_).

**Table 5:** Binding energy scores of essential oil constituents from Turmeric–black cumin combined with NOX2, NF-κβ, and mdm2 proteins.

Essential Oil Compounds	Binding Energy Score kcal/mol		
	NOX 2	NF-κβ	mdm2
α-Phellandrene	−7.1	−4.9	−6.3
*p*-Cymene	−7.1	−4.8	−5.5
*o*-Cymene	−6.9	−5.1	−5.1
*p*-Cresol	−6.5	−4.2	−5.8
*o*-Guaiacol	−5.2	−4.5	−4.4
*Trans*-4-methoxy thujane	−5.4	−4.7	−4.2
4-Methyl-1-(1-methylethyl)-3-cyclohexenol	−5.7	−5.5	−6.0
*p*-Thymol	−5.5	−5.8	−4.5
2-*epi*-α-Funebrene	−7.3	−5.6	−6.9
*Ar*-Curcumene	−8.0	−6.1	−7.1
δ-Curcumene	−7.8	−8.1	−8.5
β-Sesquiphellandrene	−7.4	−6.2	−6.8
*Ar*-Turmerol	−7.5	−5.8	−6.5
*Ar*-Turmerone	−7.5	−5.7	−6.1
α-Turmerone	−7.2	−5.3	−6.5
Curlone	−7.1	−5.7	−5.1
(*Z*)-γ-Atlantone	−7.2	−6.2	−6.2
(*Z*)-α-Atlantone	−6.9	−5.8	−5.2
(*E*)-α-Atlantone	−7.5	−5.3	−4.9
Palmitic acid	−5.8	−4.7	−4.5
Oleic acid	−5.1	−4.1	−4.3
Stearic acid	−5.5	−4.3	−4.7
Standard	L-ascorbic acid (−5.7)	Diclofenac (−5.9)	5-Fluorouracil (−4.7)

## Data Availability

Data are contained within the article.
